# Anesthetic Considerations in a High-Risk Pregnant Patient With Myelomeningocele and Urosepsis Undergoing Emergent Surgery: A Case Report

**DOI:** 10.7759/cureus.112535

**Published:** 2026-07-12

**Authors:** Maxwell Sievers, Ryan Meral, Douglas Anderson

**Affiliations:** 1 Department of Anesthesiology, University of Michigan, Ann Arbor, USA

**Keywords:** anesthesia, myelomeningocele, neuraxial anesthesia, neurogenic bladder, non-obstetric surgery, pregnancy, rocuronium, sugammadex, transaminitis, urosepsis

## Abstract

The patient was a pregnant 30-year-old gravida 6, para 0 (G6P0) woman at 22 weeks and four days (22w4d) with myelomeningocele and neurogenic bladder who presented with progressive pain, fevers, chills, and transaminitis concerning for urosepsis requiring emergent cystolithalopaxy.

Preoperative evaluation identified marked lower-extremity weakness. Anesthetic considerations included the safety of neuraxial anesthesia and concerns regarding neuromuscular blockade and reversal. Neuraxial anesthesia was avoided due to myelomeningocele, and succinylcholine was avoided due to concern for hyperkalemia. Rocuronium was used, with sugammadex administration for neuromuscular blockade reversal following consultation with obstetrics. The case proceeded uneventfully.

## Introduction

Anesthesia for pregnant patients undergoing non-obstetric surgery poses complex physiologic and pharmacologic challenges [1,2]. Additionally, underlying maternal conditions such as spinal dysraphism and neurogenic bladder with recurrent urinary tract infections elevate the risk to both mother and fetus. The presence of a myelomeningocele (the most severe and most common form of spina bifida) introduces additional considerations regarding neuraxial access, the risk of tethered cord or scarring, and altered anatomy [3-5]. Furthermore, in pregnant patients requiring neuromuscular blockade and reversal, the choice between succinylcholine, rocuronium plus neostigmine, and rocuronium plus sugammadex requires the careful weighing of maternal, fetal, and obstetric factors [1,6-8]. We present a case of a high-risk pregnant patient at 22-week gestation with myelomeningocele, neurogenic bladder, transaminitis, and urosepsis undergoing emergent cystolitholapaxy, and we discuss the anesthetic considerations and management in the context of the literature.

## Case presentation

A 30-year-old gravida 6, para 0 (G6P0) woman at 22 weeks and four days (22w4d) of gestation was admitted for the evaluation of fever, chills, worsening lower-extremity pain, pruritus, and the laboratory evidence of transaminitis. Her obstetric history was notable for multiple prior pregnancies but no viable births. She had a known history of myelomeningocele at L3-L4 that was spina bifida-associated and repaired at birth with resulting neurogenic bladder, requiring intermittent catheterization and causing frequent urinary tract infections. Although she retained sensation to light touch and painful stimuli in the lower trunk and extremities, she had long-standing lower-extremity sensory and motor deficits with very limited mobility in her lower extremities. No history of prior tethered cord release was available in the perioperative record. Laboratory evaluation on the day prior showed a white blood cell count of 18.6, elevated platelets of 654, an aspartate aminotransferase (AST) of 55, an alanine aminotransferase (ALT) of 327, and an alkaline phosphatase of 132. Complete laboratory results are shown in Table 1. She was afebrile and hemodynamically stable.

**Table 1:** Complete preoperative laboratory workup from the day prior to the procedure. WBC, white blood cell; RBC, red blood cell; HGB, hemoglobin; HCT, hematocrit; MCV, mean corpuscular volume; MCH, mean corpuscular hemoglobin; MCHC, mean corpuscular hemoglobin concentration; RDW, red cell distribution width; PLT, platelet; MPV, mean platelet volume; NRBC, nucleated red blood cell; CO_2_, carbon dioxide; AST, aspartate aminotransferase; ALT, alanine aminotransferase; eGFR, estimated glomerular filtration rate; H, data is abnormally high; L, data is abnormally low; CKD-EPI, Chronic Kidney Disease Epidemiology Collaboration

Laboratory Study	Reference Range and Units	Value
WBC Count	4.0-10.0 K/μL	18.6 (H)
RBC Count	3.90-5.00 M/μL	3.11 (L)
HGB	12.0-16.0 g/dL	9.1 (L)
HCT	36.0%-48.0%	26.9 (L)
MCV	79.0-99.0 fL	86.5
MCH	27.0-32.0 pg	29.3
MCHC	32.0-35.0 g/dL	33.8
RDW	11.5%-15.0%	16.5 (H)
PLT	150-400 K/μL	654 (H)
MPV	9.0-12.2 fL	8.9 (L)
NRBC Percentage	/100 WBC	0
Sodium	136-146 mmol/L	135 (L)
Potassium	3.5-5.1 mmol/L	3.7
Chloride	98-108 mmol/L	105
CO_2_	20-31 mmol/L	20
Urea Nitrogen	8-20 mg/dL	9
Creatinine	0.50-1.00 mg/dL	0.64
Glucose	70-180 mg/dL	118
Calcium	8.6-10.3 mg/dL	8.7
Protein	6.0-8.3 g/dL	6.3
Albumin	3.5-4.9 g/dL	3.6
AST	<34 U/L	55 (H)
ALT	10-49 U/L	327 (H)
Alkaline Phosphatase	40-116 U/L	132 (H)
Bilirubin, Total	0.2-1.2 mg/dL	0.7
eGFR, Creatinine-Based Formula (CKD-EPI 2021)	≥60 mL/minute/1.73 m^2^	122
Anion Gap	2-13 mmol/L	10

**Figure 1:**
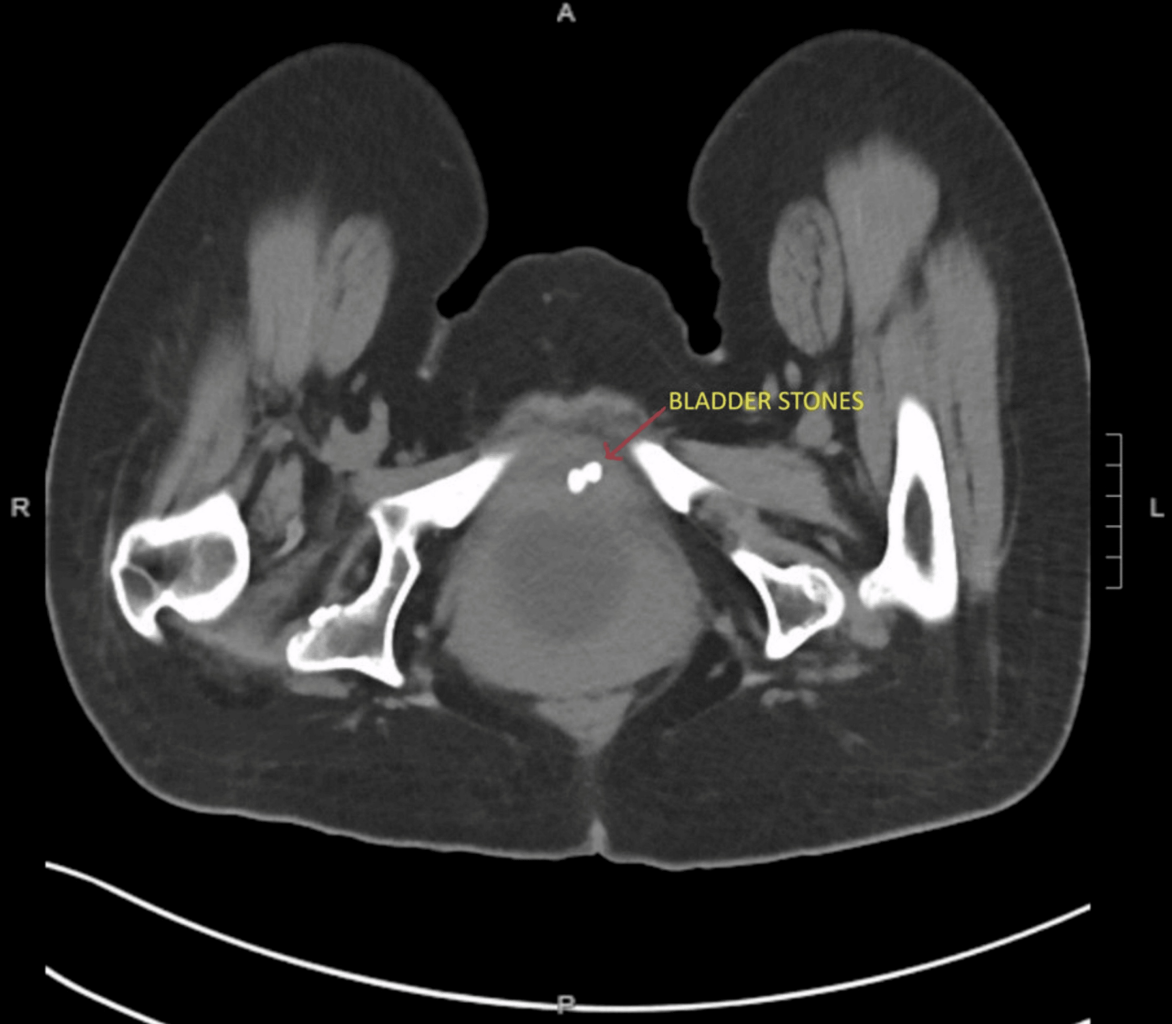
Cross-sectional image of the patient's preoperative CT scan showing two large, non-obstructing bladder stones.

Preoperatively, she was found to have *Pseudomonas aeruginosa* bacteremia in the setting of a positive urine culture. An abdominal/pelvic CT scan obtained during the evaluation of the bladder stones, which were as large as 1.1 cm (Figure 1), also included portions of the lower lumbar/sacral spine and demonstrated known dysraphism and postsurgical changes in the lumbosacral region. However, this study was not a dedicated neuraxial imaging study and did not adequately define the conus medullaris, possible tethering, postsurgical epidural scarring, or a clearly safe level for neuraxial access. No dedicated lumbar MRI was available before the emergent procedure.

**Figure 2:**
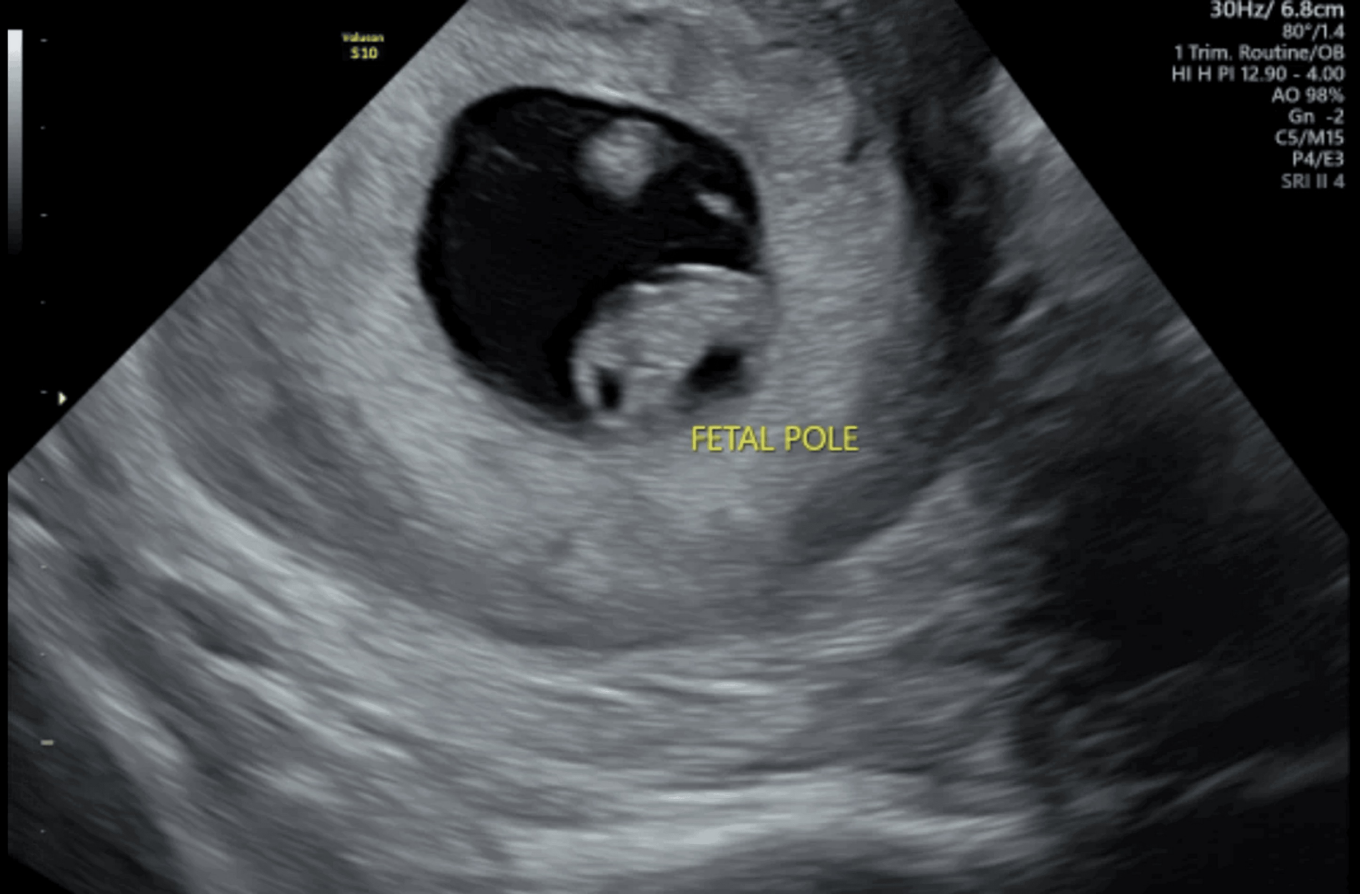
First-trimester transvaginal ultrasound showing singleton fetal pole. This image is included to show consistency in gestational dating by last menstrual period but is not meant to represent the gestational age of the fetus at the time of this case.

Obstetric ultrasound revealed a viable singleton fetus consistent with 22 weeks of gestation. Figure 2 shows an early ultrasound showing a singleton fetus with a gestational age consistent with the patient's last menstrual period.

Given the patient's bladder stones and *Pseudomonas* bacteremia refractory to catheterization and antibiotics, the urology team recommended emergent cystolitholapaxy for source control. Preoperative anesthetic evaluation identified several key issues: uncertain neuraxial anatomy in the setting of repaired myelomeningocele and significant baseline neurologic deficits, the potential risk of tethered cord or postsurgical scarring without dedicated MRI, chronic immobility and neuromuscular deficits raising concern for succinylcholine-associated hyperkalemia, gestational age requiring fetal considerations, and possible intraoperative hemodynamic instability in the setting of sepsis.

After multidisciplinary discussion with obstetrics and urology, we proceeded with general anesthesia with endotracheal intubation, rocuronium for neuromuscular blockade, and sugammadex for reversal. Standard American Society of Anesthesiologists (ASA) monitors were used with fetal monitoring before and after surgery, and the patient was maintained on scheduled antibiotics. She was positioned on the operating room table with a bump under her right hip to optimize left uterine displacement and avoid aortocaval compression.

In the operating room, anesthesia was induced with propofol, fentanyl, and rocuronium (1.2 mg/kg). A rapid-sequence induction was performed given pregnancy-related aspiration risk. Maintenance was with sevoflurane. Intraoperative hemodynamics remained stable, and estimated blood loss was minimal. At the end of the case, train-of-four monitoring showed zero twitches and a post-tetanic count of one (deep neuromuscular blockade), so 4 mg/kg sugammadex was administered. The patient was extubated uneventfully. The time from induction to extubation was 46 minutes. Postoperative fetal heart tones were reassuring. The patient was admitted to the postanesthesia care unit for monitoring, where she had stable hemodynamics. She was then transferred back to the obstetric floor.

Within one week of her procedure, her ALT normalized, and her white blood cell count decreased. During her hospitalization, she continued to experience complications related to her underlying comorbidities. At 27 weeks and four days of gestation, she developed preterm premature rupture of membranes (PPROM) and preterm labor. The obstetric team considered her postsurgical changes in the abdomen related to her prior neurogenic bladder repair and elected to perform a classical cesarean section via vertical incision. The neonate required NICU care but had no anomalies attributable to the prior surgery or anesthesia.

## Discussion

This case highlights several important anesthetic considerations in a pregnant patient undergoing non-obstetric surgery. We focus on three issues: neuraxial anesthesia in myelomeningocele, the reversal of rocuronium with sugammadex in pregnancy, and conditions prompting the avoidance of succinylcholine.

Neuraxial anesthesia in myelomeningocele

Spinal dysraphism, including myelomeningocele, presents important challenges for neuraxial anesthesia. Historically, neuraxial techniques in patients with open or repaired spinal dysraphism were approached cautiously because of abnormal bony and neural anatomy, low-lying conus or tethered cord, postsurgical scarring, altered epidural space anatomy, the unpredictable spread of local anesthetic, technical difficulty, block failure, and potential neurologic injury [3,4]. A study by Murphy et al. in 2015 reviewed 84 obstetric cases with patients who had spinal dysraphisms. They found a complication rate for epidural anesthesia to be 19 in 52 (36.5%) and for spinal or combined spinal epidural (CSE) anesthesia to be 12 in 15 (80%) [9]. However, more recent reports demonstrate that neuraxial anesthesia can be feasible in carefully selected patients, particularly when there has been antepartum planning, documentation of baseline neurologic deficits, and review of spinal imaging to identify the conus medullaris, prior surgical levels, and a safe interspace for needle or catheter placement [10,11].

In the present case, we did not regard myelomeningocele as an absolute contraindication to neuraxial anesthesia. Rather, our decision was based on the patient-specific risk-benefit assessment at the time of an emergent non-obstetric procedure. The patient had a repaired L3-L4 myelomeningocele, neurogenic bladder, chronic lower-extremity sensory and motor deficits, and very limited lower-extremity mobility. Although abdominal/pelvic CT imaging was available and included portions of the lower lumbar/sacral spine, it was not obtained to evaluate neuraxial anatomy and did not adequately define the conus medullaris, possible tethering, epidural scarring, or a safe puncture level. No dedicated lumbar MRI was available before surgery.

Because the procedure was emergent for source control in the setting of bacteremia/urosepsis, there was insufficient time to obtain dedicated spinal MRI or specialist neuroradiology/neurosurgical review. Attempting spinal or epidural anesthesia under these circumstances could have entailed multiple attempts, uncertain block reliability, possible dural puncture or intrathecal catheter placement, unpredictable anesthetic spread, or neurologic injury in a patient with substantial baseline deficits. Conversely, the planned cystolitholapaxy was expected to be short, general anesthesia allowed reliable operating conditions and airway control, and succinylcholine could be avoided by using rocuronium with quantitative neuromuscular monitoring and sugammadex reversal. Therefore, while acknowledging that neuraxial anesthesia may be appropriate in selected patients with spinal dysraphism after adequate imaging and planning, we judged that general anesthesia offered the most controlled and prudent approach in this emergent setting.

Reversal of rocuronium in pregnancy (sugammadex versus neostigmine)

In pregnant patients undergoing non-obstetric surgery, the choice of neuromuscular blockade and reversal warrants the consideration of maternal-fetal physiology, placental drug transfer, and the need for reliable reversal [1,6-8]. The depolarizing agent, succinylcholine, has long been the standard for rapid-sequence induction in pregnant patients but carries well-described risks; rocuronium offers an alternative. In pregnancy, however, this decision is complicated by the need for a reversal agent. Historically, a combination of a cholinesterase inhibitor and a complementary antimuscarinic agent, neostigmine and glycopyrrolate, respectively, has been used, yet sugammadex, a cyclodextrin-derived relaxant binding agent, has demonstrated superior efficacy as measured by the speed of onset, reduced residual paralysis, bradycardia, and overall adverse effects [12]. Animal and in vitro studies have suggested that sugammadex may bind endogenous progesterone, potentially reducing circulating levels [6,8]. As progesterone is essential for maintaining uterine quiescence, theoretical concerns exist regarding miscarriage or preterm labor. In 2019, the Society for Obstetric Anesthesia and Perinatology (SOAP) recommended "avoid or use with caution" in near-term pregnancy, yet controversy exists. Limited clinical data have not demonstrated adverse outcomes, and despite warnings against sugammadex use in pregnant patients from Merck and SOAP and the lack of FDA approval, a growing number of case reports are being published regarding its use in pregnant patients [1,2,7]. In this case, sugammadex was chosen following multidisciplinary consultation and provided rapid, reliable reversal without residual blockade.

With sugammadex administration, vigilance is warranted in monitoring for severe allergic reactions. In one analysis of 33 published cases, the incidence of sugammadex anaphylaxis was estimated between one in 2500 and one in 10000 administrations, with 92% having symptoms within five minutes, most commonly hypotension and tachycardia [13].

Conditions to avoid succinylcholine

Succinylcholine remains widely used for rapid-sequence induction; however, its use is contraindicated or relatively contraindicated in a number of conditions, including hyperkalemia, neuromuscular disorders, burns, extensive trauma, immobilization, spinal cord injury, denervation syndromes, and conditions causing the upregulation of extrajunctional acetylcholine receptors [14]. In our patient, long-standing neuromuscular deficits from myelomeningocele created concern for the risk of exaggerated hyperkalemia after succinylcholine administration, making rocuronium preferable.

Perioperative management of pregnant patients undergoing non-obstetric surgery

Pregnant patients undergoing non-obstetric surgery require coordinated planning among numerous specialties, including obstetrics, anesthesia, surgery, and neonatology. Key considerations include maternal physiologic changes (increased cardiac output, decreased functional residual capacity, and increased aspiration risk), fetal monitoring and gestational age considerations, the timing of surgery (ideally delaying until after the first trimester), maternal medications, and positioning [15]. In emergent settings (as in this case), the risk-benefit analysis must weigh maternal indications against fetal risk. In our case, maternal bacteremia mandated surgery. Fetal monitoring (pre- and postprocedural cardiotocography) was performed. Intraoperative continuous fetal monitoring was not utilized given the gestational age. Positioning included left uterine tilt to avoid aortocaval compression, and maternal oxygenation and hemodynamic stability were prioritized.

Limitations and lessons

While outcomes were favorable, evidence remains limited for sugammadex use in pregnancy and neuraxial techniques in spinal dysraphism. Multidisciplinary planning and neuromuscular monitoring are essential. In hindsight, the imaging of the spinal anatomy (MRI) might further inform neuraxial feasibility, although it was not practical in this emergent setting. A causal relationship between her prior anesthetic and subsequent PPROM cannot be determined.

## Conclusions

This case illustrates the complex interplay of maternal anatomy, obstetric considerations, pharmacologic choices, and perioperative planning. In high-risk pregnant patients such as these, general anesthesia with rocuronium and sugammadex may be a viable alternative when neuraxial anesthesia is not feasible and succinylcholine is contraindicated. Multidisciplinary coordination, neuromuscular blockade monitoring, and attention to fetal considerations remain essential. Additional research is needed to better define the safety of sugammadex use in pregnancy and of neuraxial techniques in patients with spinal dysraphism.
